# Traits, Trends, and Trajectory of Tween and Teen Cyberbullies

**DOI:** 10.7759/cureus.9738

**Published:** 2020-08-14

**Authors:** Farah Khan, Therese Limbana, Tehrim Zahid, Noha Eskander, Nusrat Jahan

**Affiliations:** 1 Psychiatry, California Institute of Behavioral Neurosciences & Psychology, Fairfield, USA; 2 Internal Medicine, California Institute of Behavioral Neurosciences & Psychology, Fairfield, USA

**Keywords:** cyberbully, elementary school cyberbully, tween cyberbully, adolescent cyberbully, cyberaggression, cyberbullying, cyberbullying perpetrator

## Abstract

Digital communication has revolutionized the way children interact and maintain social relations. However, not every tween (8-12 years) or teen (13-18 years) is able to take full advantage of digital media and may cross personal and social boundaries causing distress, mostly to their own friends at school and beyond. This results in adverse health effects for both the cyberbullying perpetrator and the victim. Articles reviewed on elementary school children and adolescents, collected from two different databases, showed that the number of elementary school kids using smartphones has more than doubled in the past few years. Given this rise, the risk of cyberbullying has also increased. Not all elementary school kids have the required media literacy to understand that their friends have equal rights in the virtual world as they do in the schoolyard. Regardless, they still carry a smartphone with data, use computers, and other electronic media to bully, embarrass, exclude, or humiliate others, often through social networking sites. Moving from tweens to teens seems to worsen the cyberbully behavior and choices, with middle school kids facing the highest cyberbullying incidents followed by high school kids and then the elementary school kids. The anonymity of cyberspace and the perceived lack of consequences seems to embolden the cyberbully. Identifying the mindset of a cyberbully and those at high risk of becoming a cyberbully can help target intervention efforts where they are needed the most and prevent cyberbullying.

## Introduction and background

Access to digital media has revolutionized communication, changed social interactions, and presented new challenges everywhere for children, parents, teachers, researchers, and policymakers in the form of cyberbullying. Cyberbullying is a deliberate act of offense meant to abuse another person using electronic gadgets [1]. Tweens and teens used information communication technology like computers and smartphones to bully, embarrass, exclude, or humiliate others. This aggression is performed using media such as online games, social media forums, online chat-rooms, instant messaging applications, video chats, and text messaging, etc. [2]. The online realm is perceived as anonymous and invisible, and it offers a lack of personal boundaries. Punishment, repercussions, and consequences of these actions are also thought of as slim in the virtual world. This sets precedence to toxic online disinhibition resulting in hatred, threats, rude language use, lack of empathy, and lessened self-control [3]. The lifetime experiences of cyberbullying in the proportion of people have more than doubled (18% to 37%) from 2007 to 2019, and this issue has become a major public health problem affecting tweens and teens [4,5]. 

Most research and literacy on cyberbullying is focused on adolescents in middle and high school. Seldom has research and cyber literature been focussed on pre-tweens (before 8 years) or tweens (8-12 years) in elementary school, where kids first get access to digital media [6]. Hence, very scarce information exists with regard to cyberbullying among tweens and pre-tweens especially from the perspective of the cyberbully. When does a child first become a cyberbully? When does cyberbullying start in elementary school and how does it evolve? Was the probullying attitude rewarding or were the prodefending attitudes not favorable? This article will try to analyze if the research has answered these pending and crucial questions. 

The Common Sense Census study explores how media use and digital trends have evolved over time among tweens and teens. A survey that follows the tweens (8-12 years) and teens (13-18 years) came out with a recent report in 2019 with the results of the amount of daily screen use. Tweens were spending an average of 4.44 hours per day, while teens were spending 7.22 hours per day on screens time unrelated to school and homework. The study found that 56% of tweens and 69% of teens watched online videos every day. Approximately 19% of 8-year-olds and 69% of 12-year-olds now own a smartphone [7]. The use of digital media has doubled in a span of few years in these age groups resulting in an increased risk of cyberbullying [8]. 

Even though it is seldom studied by research, there is an association between a cyberbully/perpetrator and adverse outcomes. A review of the personality traits, trends, and behavior of the cyberbully can help in early intervention and prevention of cyberbullying in the future. These determinants are important as they could change the trajectory of the cyberbully early on with timely intervention, empower parents and the school administration with the tools to appropriately handle these issues, enable researchers to make profiles of cyberbullies, and provide policymakers and school administration with vital knowledge about the students at high risk to whom intervention efforts should be targeted. 

Methods 

Articles were searched in two different databases: PubMed and Google Scholar. The regular keywords used can be seen in Table 1.

**Table 1:** The following regular keywords were used for data collection.

Keywords
Cyberbullying perpetrator
Cyberbully
Cyberbullying
Elementary school cyberbully
Tween cyberbully
Adolescent cyberbully
Cyberaggression

PubMed Database

Studies were selected and reviewed after applying the mentioned inclusion and exclusion criteria below on PubMed. 

Inclusion criteria: 

Ages: Less than 18 years. 

Gender: Both female and male. 

Language: All articles were in English. 

Age of literature: All articles were published within the last 10 years. 

Exclusion criteria: 

Ages: Not more than 18 years. 

Language: Non-English languages. 

The articles selected from PubMed were broken down as seen in Table 2: 

**Table 2:** Article breakdown.

Records	Quantity
Total records	103
Articles selected	41
Number of full articles	20
Abstracts only	21
Articles removed	62
Duplicates	0

Google Scholar Database 

Nine full articles were collected manually from Google Scholar using the same search criteria based on the most recently published literature, title, and abstract content. 

Results 

After applying the inclusion/exclusion criteria the following number of articles on PubMed and Google Scholar were found. 

Regular keywords: Cyberbullying perpetrator, Tween cyberbully, Adolescent cyberbully, Cyberbully, Perpetrator traits, Elementary school cyberbully, Gender stereotype traits, Cyberaggression. 

Database 1: PubMed 

The total number of articles selected after review and a refined search was 41 as they fit the selection criteria. 

The articles removed were not included for the lack of relevant data. 

**Figure 1:**
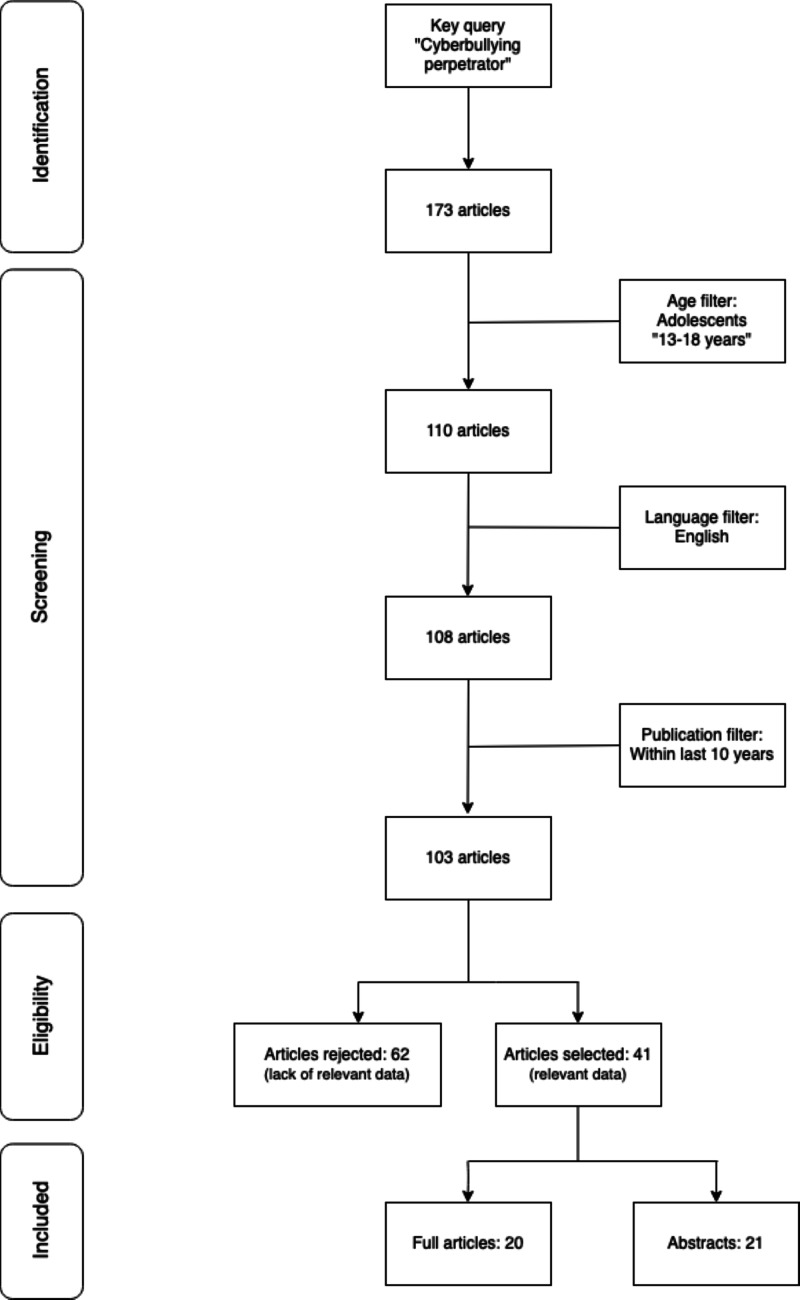
Inclusion/exclusion criteria applied for data collection from PubMed.

The flowchart seen in Figure 1 shows the starting keywords used and the number of articles obtained on PubMed for literature search with the applied filters. Finally, the total number of used articles is displayed alongside those which were not selected. 

Database 2: Google Scholar 

Nine full articles were then obtained manually from Google Scholar. The articles were selected based on the most recently published literature, title, and abstract content based on the inclusion/exclusion filters. Five full articles were chosen for cyberbullying in elementary school, two full articles were chosen for gender disparities in cyberbullying, and one full article was for cyberbullying interventions. 

The final number of articles selected from PubMed was 41, and 9 full articles from Google Scholar. The total number of articles from both databases was 50. One article from Google Scholar was gray literature, and hence not used. The maximum number of subjects in a study was 162,034 middle school kids from one school district. 

## Review

Discussion 

Understanding the mindset, traits, and circumstances of the cyberbully, as well as those of children at high-risk of becoming cyberbullies, can help with timely interventions and prevention of cyberbullying, thereby building safer communities. Cyberbullying is an expression of violence using electronic media that can cause adverse mental health effects. Cyberbullying involves an individual, or a group of perpetrators, a victim, and potentially online bystanders. It is intentional and causes psychological distress [9]. The mean age of victimization is around 14 years when adolescents spend large amounts of time on their mobile phones and social networking sites [10,11]. Mocking and spreading rumors about others is the most popular form of cyberbullying. Cyberbullying trends are highest in middle schools followed by high schools, and finally by elementary schools [12]. Facebook was overwhelmingly the most commonplace place for cyberbullying to occur among teens [13]. A study found that flaming or "online fight" was associated with both perpetration and victimization of cyberbullying, and increased risk of perpetration only was seen in players with online game addiction [14]. The cyberbully has a degree of anonymity and lack of adult supervision, can reach their victim(s) at any time and have the ability to affect greater audience, and damage reputation(s) which makes it more dangerous than traditional bullying [11,15]. This causes distress and a greater sense of insecurity and lack of control, leading to hopelessness amongst the cyber victims [15]. Using electronic media like internet, text messaging, web cameras, posting personal information, and harassing others online were linked to cyberbullying. Cyberharassment was often perpetrated via phone calls, text messaging, chat rooms, through pictures or video clips sent via mobile phones, emails, or websites. Cyberharassment behaviors are the use of abusive words, saying mean things or making fun of the victim, solicitations for relationships or sex, and spreading rumors about the victim [2]. Childhood bullies are of two types: those seeking social status and those going after vulnerable victims to exercise their power and control. The vulnerable victims include peers who are without friends, or are disabled, or if coming from broken families and those without other support systems. A study involving more than 16,000 American public and private school students from grades 6 to 10 concluded bullies inspired by social status mostly target their friends and other more popular students at school [16].

Theories

Developmental theory, or social learning theory, states that children's behavior can be predicted by their attitudes, perceptions, and self-efficacy beliefs, whereas other studies focusing on increased cyberbullying behavior found a relationship between beliefs, attitudes, and behavior with acceptable attitudes about bullying and aggression [17,18]. One more study on the predictors of cyberbullying involvement states low cyberbullying perpetration rates in students who had higher levels of pro-victim attitudes [19]. The theory of planned behavior concludes that intentions determine behavior [20]. The theory of planned behavior contributes to a structure for predicting adolescent cyberbullying perpetration through interventions primarily focused on changing normative and acceptable beliefs toward cyberbullying into negative attitudes. Prevention programs should sustain that many adolescents prevail in skeptical attitudes about cyberbullying. One possible explanation of high-risk cyberbullying could be that adolescents who witness online hate or believe it is acceptable among peers will normalize that behavior and be more likely to perpetrate online hate.

Prospective risk factors for cyberbullying 

High-Risk Predictors 

Studies concluded that cyberbullies are mostly male, while victims are more likely to be females and sexual minorities [5]. However, other studies show no significant gender difference in adolescents either as aggressors or as victims [21,22]. Cyberbullies usually have low academic performance which may destroy their self-esteem, making them less pro-social and increase the frustration resulting in aggressive behaviors and cyberbullying [23]. They seem to get trapped in a negative school climate with low peer support and end up with peers who share dangerous values. These values include a moral approval of bullying, antisocial behaviors, and normalization of violence [24]. This aggressive behavior may make them unpopular with peers resulting in exclusion and discrimination from peers. Bullies who pick on vulnerable victims are anxious or depressed and less popular themselves. Cyberbullies have low levels of empathy, high self-esteem, and frequently consume violent media like watching excessive violence on television and play violent video games, which desensitize them to aggression and violence [25]. Traditional bullies at school are three times as likely to engage in cyberbullying [26]. The inability to read non-verbal cues and alexithymia, or the inability to express emotions, may also result in becoming a cyberbully [27,28]. Cyber victims are also at increased risk of becoming cyberbullies. For cyber victims, the risk of perpetration of both sexual and psychological behaviors increases. The sexual cyberbullying prevalence is correlated with being male and the experience of psychological and sexual aggression online. Females mostly practice psychological cyberbullying perpetration. Having a poor emotional bond with a caregiver, family conflict, physical aggression, and bullying are established predictors of youth violence and aggression [24,29]. Cyberbullies may come from a family background where rules and boundaries are unclear putting them at high risk for violent and antisocial behaviors [24]. Some cyberbullies have a low level of access to supervision by adults, and it could be possible that parents/guardians of the cyberbullies are insufficiently trained, not confident or new to technology to effectively monitor their child's use of information communication technology [30]. Other factors such as a controlling parenting style as well as an inconsistent internet-mediation style were associated with a higher prevalence of adolescent involvement in cyberbullying as victims and as perpetrators [31]. Studies have revealed that family members have the potential to change the trajectory by discouraging, exacerbating, or by interfering with cyberbullying [32]. For girls, cyberbullying involvement, both as cyberbullies and as victims, could be due to intense cyber socialization and a greater amount of online contact with strangers. For boys, higher levels of victimization were predicted by increased exposure to antisocial media content over time [33]. Students using the internet more frequently are significantly at risk of being cyberbullying perpetrators, victims, and perpetrators-victims. Cyberbullies may carry guns for intimidation and victims for self-protection [34]. Studies have found that over 85% of cyberbullies are also involved in traditional bullying [8]. Traditional bullies at school are three times as likely to engage in cyberbullying and a victim of traditional bullying is linked with being a cyberbully [29]. Many cyberbullies and cyber victims are also traditional bullying perpetrators and victims, respectively [35]. 

Low-Risk Predictors 

Students who are connected to the school and attend the institution in a healthy and positive environment, those with strong parental support, and healthy social connections with teachers show low potential to be a cyberbully [18]. Parental restrictive mediation with emotional support was associated with reductions in adolescent Internet addiction and cyberbullying. 

*Tweens* 

Very few studies have investigated cyberbullying in elementary school. A study in the USA concluded that about a fifth of three- to six-year-olds had a computer in their bedrooms [36]. Most cyber victims reported bullying through online games. The bullying often starts in school and is continued at home and the victims often know the cyberbully from school [8,37]. About 38% of cyber victimized children knew the identity of the cyberbully and almost 50% did not tell anyone about the incident [38]. Tweens would propose a victim of cyberbullying to tell someone and the endorsed coping strategy for victims was to tell someone [6]. 

*Gender* 

Male adolescent cyberbullies tend to externalize and engaged in physically aggressive forms of bullying, whereas females tend to internalize and relied on verbal and social cyberaggression. Adolescent male cyberbullies were at higher risk for tobacco smoking; however, those who were only cyber victims or victims/perpetrators were at higher risk for alcohol drinking [39]. Students (both boys and girls) with more feminine traits were more committed to cyber relational aggression through social networking sites and mobile phones. On the other hand, those adolescents, both boys and girls, with masculine traits indulged more in hacking and expressed cyber aggression through online games using all forms of technology when compared with those adolescents who reported feminine traits [40]. 

Support Systems 

Social support works as a protective shield in stressful situations by offering a soft place to land on in stressful times for youth. Adolescents identified sharing the bullying situation with a friend as a helpful coping strategy [41]. Strong family support where open communication is practiced between family members and emotional support provided with moral guidance along with healthy social support from teachers can validate and reinforce positive behavior and values in kids. These can work as protective factors by strengthening self-esteem and building resilience in tweens and teens. The authoritarian parenting style providing warmth and support dimension was associated with less supportive attitudes toward cyberbullying and lower levels of cyberbullying in emerging adulthood [42]. 

Types of Cyberbullying 

A form of cyberbullying where personal information is gathered and released to the public is called doxing. It violates the victim’s privacy and makes them more vulnerable to future harassment. Girls are more likely to conduct social doxing where their target was to obtain social information, whereas boys mostly engage in hostile doxing aimed at retrieving personally identifiable information and information on others' current living situations. Cyberbullies typically engage in doxing with the malicious intent to humiliate, threaten, intimidate, or punish a person. By disclosing victims’ personal information, doxing cyberbullies encourage others to participate in online harassment. Different types of cyberbullying are seen in Table 3.

**Table 3:** Various types of cyberbullying. Source: Kowalski et al. (2014) [43].

Type	Description
Flaming	Online fight
Harassment	Repetitive, offensive messages sent to a target
Outing and trickery	Discovering personal information about someone and then electronically sharing that information without the individual's permission
Exclusion	Blocking an individual from buddy lists or other electronic communications
Impersonation	Pretending to be the victim and electronically communicating negatively or inappropriately with others as if the information is coming from the victim
Cyberstalking	Using electronic communication to stalk someone by sending repeated threatening messages
Sexting	Sending nude/inappropriate photos of another person without that individual's consent

Cyber Victims 

Comprehending who is targeted by cyberbullies from vulnerable groups will help permeate intervention activities. Victims are targeted for different reasons including their religion, gender, physical appearance, race/ethnicity, disability, and sexual orientation [44]. Students who used the internet at least for three hours per day, those who used webcams, and instant messaging are more likely to have been cyber victimized at least seven times during the previous year [8]. 

*Effects* 

Cyberbullying causes adverse health effects on both the victim and the bully themselves. It is associated with negative outcomes such as an increased risk of depression, social anxiety, substance abuse, and violent behavior [45-47]. They display a higher incidence of suicidality, unsafe sexual behavior, and social and psychological disturbances in life compared to non-victims and non-perpetrators [23,35]. Cyber victims and cyberbullies have more emotional and psychosomatic problems, social difficulties, and feel unsafe and uncared for in school. Cyberbullies experienced more physical symptoms, while cyber victims dealt with more psychological distress [42]. The adolescents who have been in the cyberbully-victim group exhibit the highest levels of depressive symptoms, and the lowest levels of family support and subjective well-being [41]. 

Long-Term Consequences 

Cyberbullies face long-term consequences including alcohol/drug use, dropping out of school, criminal convictions, early sexual activity, and being emotionally and physically abusive to others as adults. Both the cyberbully and the cyber victim tend to engage in suicidal ideation and are more at risk of committing suicide. Other long-term consequences for both cyberbully and cyber victim are shown in Table 4.

**Table 4:** Consequences for cyberbullies and their victims. Adapted from Eisenberg and Aalsma (2005) [48].

Consequences
Suicide/suicidal ideation (bullies and victims)
Poor physical health/somatic complaints
Deterioration of chronic health conditions
Depression
Low self-esteem
School absenteeism
Violence-related behaviors
Substance use (bullies and victims)
Academic failure (bullies and victims)
Externalizing problems (bullies and victims)
Internalizing problems

Recommendations 

The following proposals are for clinicians, families, schools, communities, future researchers, policymakers, and social media platforms to help both cyber victims and cyberbullies in effectively changing their trends and trajectory, thereby providing a healthy childhood, building safe virtual zones for kids, and ultimately stronger, safer, and better communities. 

1) Apart from screening for cyberbullying, all primary care physicians, pediatricians, and mental health professionals should support and advocate for cyber victims starting at a pre-tween age. At this same time, identifying cyberbullies and guiding them to appropriate resources is just as important. Health care professionals should emphasize open communication in the family and positive parenting. Families should be linked with appropriate intervention strategies and followed through. Links available on StopBullying.gov [49].

2) The educative and social practices should focus on the responsible and safe use of information and communication technology so that the tweens and teens are able to make full use of the cyberspace while learning to respect diversity and navigate through risks and potential cyber aggression. All possible channels, including the family, school, community, and media, should be used to convey this message and also as support systems when needed. 

3) Prevention and early intervention programs against cyberbullying should be introduced at elementary school level aimed at resilience building, moral and positive value promotion, age-appropriate emotional skills training (lexicon and expression of positive and negative feelings), social skills development, conflict resolution skills, democratic values, and media literacy programs. These ideas will help tweens and teens understand that basic human rights are universal and lack boundaries. 

4) New programs should be designed and promoted to deal with victim empathy, critical self-monitoring, self-reflection, and self-control as well as teach tweens and teens to recognize social cues that might reduce online disinhibition effects. 

5) Digital literacy, prevention, and intervention programs should be all inclusive and target individuals at high risk of cyberbullying. These programs should be introduced early on when kids first get access to digital media at the elementary school level. Digital literacy should also include on how electronic bystanders may appropriately intervene. 

6) Multifaceted intervention programs for cyberbullies and victims should be developed in consideration of cultural, gender, sexual identity, religion, and other individualized factors across social online platforms where most cyberbullying occurs. 

7) Parents/guardians should set time limits, supervise internet activity, and set up parental controls on media devices. New policies should be put into effect that will implement efficient reporting of inappropriate content and at the same time require social media platforms to identify and hide or rank such content lower in the news feeds. 

8) Promote a healthy lifestyle with a balanced diet, exercise, and more sleep which reduces stress and builds confidence and resilience in tweens/teens. 

9) Research examining the effectiveness of the current antibullying laws and policies is also recommended. Designing activities based on sound scientific evidence will help protect the tweens/teens and help them grow confident and healthy as exceptional members of society. 

10) Antibullying programs and protocols should address the needs of both cyber victims and cyberbullies. 

11) More investigation is required to interpret the mindset of a cyberbully in their pre-tween and tween years. It will be of great help to identify the circumstances and choices of their actions through personal interviews of cyberbullies. This can further guide individualized interventions for long-term results.

## Conclusions

Studies that were reviewed did not answer definitively when cyberbullying starts or how it evolves. However, current literature reviews led to the understanding that cyberbullying begins in the pre-tweens, long before the adolescent age that the majority of studies focus on. More longitudinal studies on pre-tweens and tweens may shed light on this area and help us to understand how and when cyberbullying starts and evolves in elementary school. Research studies conducted when pre-tweens and tweens are first exposed to electronic communication devices and their immediate behaviors after may give a better understanding of the genesis of a cyberbully and perhaps answer if pro-bullying attitude is more rewarding or pro-defending attitudes not favorable. Qualitative research on the origin will reveal a deeper understanding of the cyberbullying problem in pre-tweens, tweens, and teens. Compiling data from those involved in cyberbullying would help to interpret the quantitative results and open new avenues for the study and intervention of cyberbullying. 
